# *UANanoDock*: A Web-Based *UnitedAtom* Multiscale Nanodocking Tool for Predicting Protein Adsorption onto Nanoparticles

**DOI:** 10.1021/acs.jcim.4c02292

**Published:** 2025-03-25

**Authors:** Julia Subbotina, Panagiotis D. Kolokathis, Andreas Tsoumanis, Nikolaos K. Sidiropoulos, Ian Rouse, Iseult Lynch, Vladimir Lobaskin, Antreas Afantitis

**Affiliations:** † School of Physics, University College Dublin, Dublin 4 D04 V1W8, Ireland; ‡ NovaMechanics MIKE, Piraeus 18545, Greece; § Entelos Institute, Larnaca 6059, Cyprus; ∥ NovaMechanics, Ltd., Nicosia 1070, Cyprus; ⊥ School of Geography, Earth and Environmental Sciences, University of Birmingham, Edgbaston, Birmingham B15 2TT, United Kingdom

## Abstract

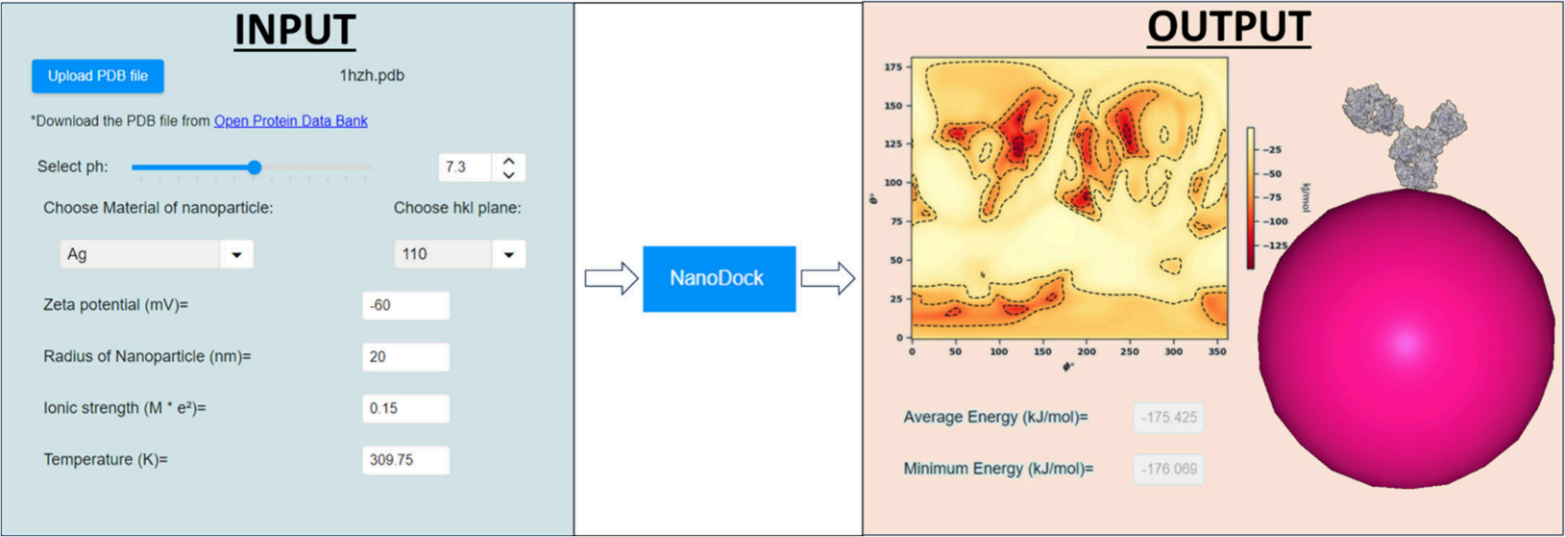

*UANanoDock* is a web-based application with a graphical user interface designed for modeling protein–nanomaterial interactions, accessible via the Enalos Cloud Platform (https://www.enaloscloud.novamechanics.com/compsafenano/uananodock/). The application’s foundation lies in the UnitedAtom multiscale model, previously reported for predicting the adsorption energies of biopolymers and small molecules onto nanoparticles (NPs). *UANanoDock* offers insights into optimal protein orientations when bound to spherical NP surfaces, considering factors such as material type, NP radius, surface potential, and amino acid (AA) ionization states at specific pH levels. The tool’s computational time is determined solely by the protein’s AA count, regardless of NP size. With its efficiency (e.g., approximately 60 s processing time for a 1331 AA protein) and versatility (accommodating any protein with a standard AA sequence in PDB format), *UANanoDock* serves as a prescreening tool for identifying proteins likely to adsorb onto NP surfaces. An illustration of *UANanoDock*’s utility is provided, demonstrating its application in the rational design of immunoassays by determining the preferred orientation of the immunoglobulin G (IgG) antibody adsorbed on Ag NPs.

## Introduction

Biomedical nanotechnology applications, including targeted drug delivery, bioimaging, and biosensors, extensively utilize nanoparticles (NPs).^1^ The high costs and time-consuming nature of *in vitro* experiments, coupled with ethical concerns surrounding *in vivo* studies due to potential harm to living organisms, have created a growing demand for *in silico* tools. These computational methods aim to replace traditional experiments in identifying NP’s biomedical applications. Although various computational approaches have been developed to examine protein adsorption on material surfaces,^2^ most face challenges balancing model dimensionality and computing efficiency.

For example, protein adsorption can be studied with the use of molecular dynamics (MD) simulations, and such cases were reported for a limited number of proteins and NPs with preset parameters.^2−6^ Yet, considering that the human proteome consists of an estimated minimum of 0.62 million protein species^7^ with more than 60,000 human proteins having a crystal structure reported in the RCSB database,^8,9^ studying their interactions for possible pharmaceutical nanoformulations varying in shape, sizes, and surface chemistry with brute force MD simulations become unfeasible. To close this gap, we have earlier introduced *UnitedAtom* methodology^10−13^ that can prescreen large protein data sets for their affinity to numerous types of NPs. While the *UnitedAtom* multiscale model is extremely fast and reliable, a simple web application that allows nonexpert users to apply this method for quick one-protein simulation without the need to install software is missing. *UANanoDock* aspires to fill this gap through the provision of a web-based graphical user interface (GUI) and its public accessibility via the *Enalos Cloud Platform* at https://www.enaloscloud.novamechanics.com/compsafenano/uananodock/, where other nanoinformatics tools (e.g., molecular builders, machine and deep learning models, exposure models, etc.) are also hosted.^14−16^ The coupling of *UANanoDock* outputs with the *Enalos Cloud Platform* nanoinformatics toolkit through application programming interface (API) calls can further lead to the creation of faster and more reliable machine-learning-based predictive models of the cytotoxicity of the NPs.

The *UANanoDock* software is capable of estimating the adsorption energy hierarchy of proteins on NP surfaces as well as the spatial configurations of protein–NP complexes. Results generated by *UANanoDock* can be further utilized in the desktop version of the *UnitedAtom* simulations. Comprehensive instructions for using *UANanoDock* are provided in the shared tutorial. To demonstrate the practical application of *UANanoDock*, here we examine a case study involving the rational design of immunoassays based on the IgG1 antibody.^17^

### Theoretical Concepts of Nanodocking Functionality of the *UnitedAtom* software

**Figure 1:**
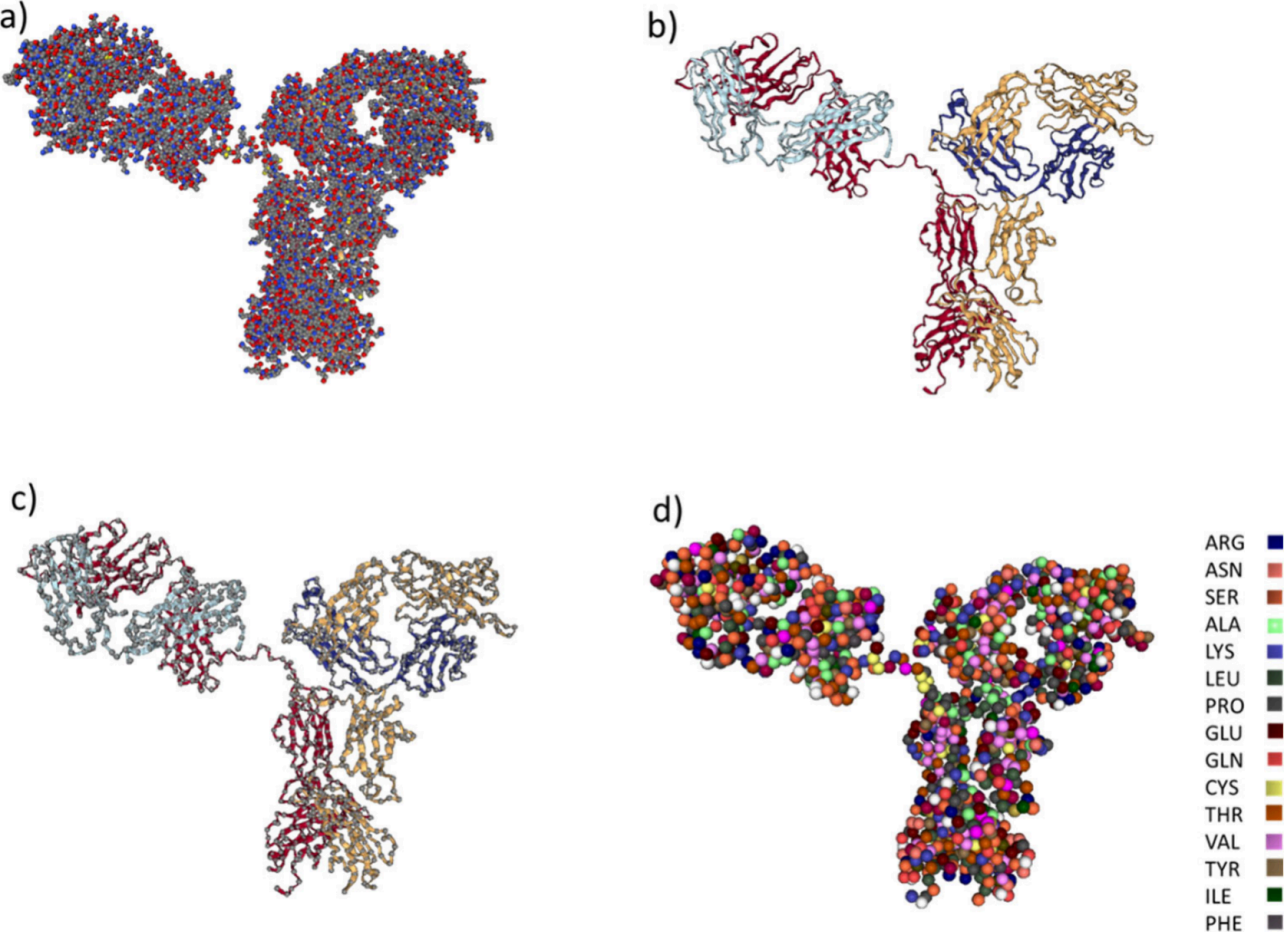
Protein coarse-graining in the *UnitedAtom* model. (a) Ball and stick representation of the atomistic IgG model. Gray, red, blue, and yellow colored atoms illustrate carbon, oxygen, nitrogen, and sulfur atoms, respectively. Hydrogen atoms have been omitted. (b) Ribbon representation for each of the four polypeptide chains of the all-atom model of IgG antibody. (c) Alpha-carbon atoms (i.e., carbon atoms connect the amino to the carboxyl group) represented with gray colors connected with ribbons. (d) CG representation of the polypeptide chains after replacing all AAs with beads centered at the position of AA alpha-carbons.

The core concept of the *UANanoDock* web tool is based on the open-source, publicly available *UnitedAtom* software tool implemented in the *NPCoronaPredict* package.^13^ The *UnitedAtom* approach is based on the coarse-grained (CG) representation of the bio-nano interface and models the interactions between protein and nanomaterials within a multiscale approach with an accelerated algorithm using precalculated interaction potentials. The *UnitedAtom* model defines proteins and nanomaterials as rigid bodies. The proteins are composed of CG amino acid (AA) beads, where each bead represents one AA residue, with the largest component of the potential representing the interaction between the side chain and the surface of the NP (Figure 1d). The coordinates of the alpha-carbon atoms of the AAs in the atomistic model of the protein, which are illustrated in Figure 1c with a gray color, are assigned to the origins of the AA beads in the CG model.

NPs are treated as homogeneous or composite bodies with a desired shape (e.g., spheres, cubes, etc.) with a specified surface potential (or **ζ**-potential) and the spatial dimensions are defined by geometric parameter *R* (e.g., *R* is the particle radius in the case of spherical NPs). The chemical composition of nanomaterial bead(s) is set by two groups of parameters for “nanomaterial - AA” interactions: Hamaker constants and short-range noncovalent interaction potentials in tabulated form. The need for two sets arises from the multiscale character of the *UnitedAtom* model which only considers noncovalent interactions between biopolymers and nanomaterials (*physisorption*). The Hamaker approach^18^ is used to describe implicitly van der Waals interactions at a long-range beyond the cutoff distance *r*_*cutoff*_ (typically the cutoff is set to 1.0 or 1.2 nm depending on the force field (FF) used). The interactions at small distances *r* < *r*_*cutoff*_ are modeled explicitly with the atomistic representation of bio-nano interface by a set of surface–surface distance-dependent potentials of mean force precalculated by enhanced sampling MD atomistic simulations for each CG AA bead-nanomaterial pair^11,19^ (see Note S1 in Supporting Information for additional information on methodology of obtaining these potentials).

**Figure 2:**
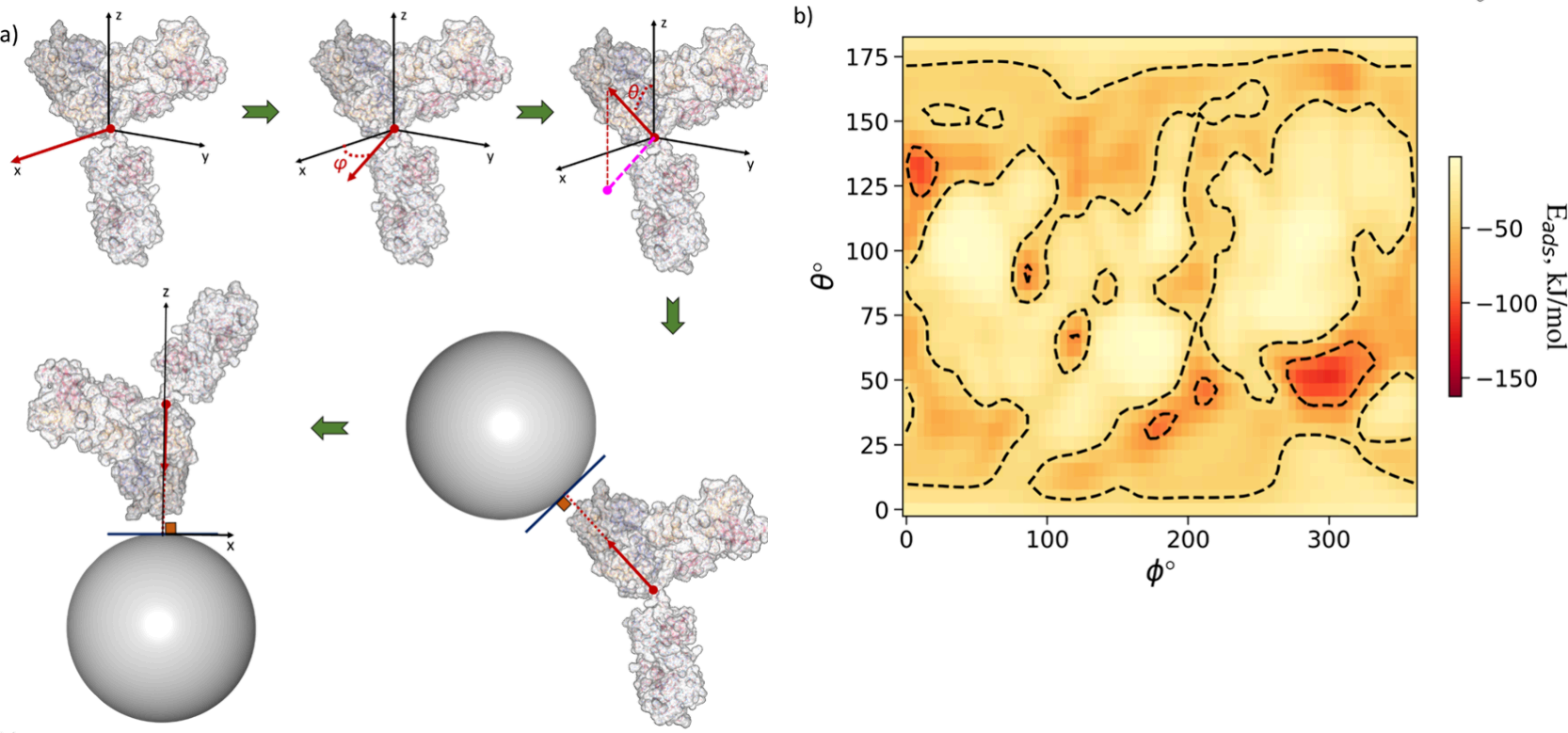
(a) Definition of ϕ and θ angles, the rotational coordinates of *UANanoDock* adsorption energy maps. The ϕ and θ angles correspond to an initial vector in the protein’s frame of reference shown in this figure which is rotated such that this vector faces toward the surface of the NP when the protein COM is at a position (0, 0, *z*) relative to the NP. This transformation is sequential with the first rotation of the protein by −ϕ around the *z*-axis, followed by 180° −θ rotation around the *y*-axis. Note that initially (at [ϕ,θ] = [0°,0°]) the protein is aligned to the principal axis with the longest axis along *z* and the second longest along *y*-axis. The single spherical bead representation of NPs is the only NP representation available in the current release of the web tool. For more complex NP constructs (e.g., PEG-coated, alloys, etc.), it is advised to use the desktop version of the *UnitedAtom* model. (b) The adsorption energy heatmap per orientation of the IgG antibody on the NP’s plane with Miller indices *hkl* equal to 111 of a silver NP having its radius equal to 15 nm and its surface potential equal to −20 mV while the ionic strength is equal to 0.15Me^2^ and the temperature is 309.75 K. The adsorption energies per orientation have been calculated from a Boltzmann average of the potential energies that correspond to a range of distances for the same IgG orientation with angular resolution δ = 5° and bin sampling *N*_bin_ = 6. The distances of the closest bead from the NP’s surface start from zero and reaching 2.0 nm beyond which are negligible.

The adsorption process is modeled as the protein’s gradual movement along a vector connecting the COM of NP and COM of protein. Consider that this vector is aligned to the *z*-direction of the Cartesian coordinate system. In this system, a spherical CG NP bead with a radius *R*_*NP*_ is fixed in the origin, and the protein can rotate and move along *z*-direction. The total interaction potential between a NP and the protein as a whole positioned and oriented at some point *A* near the NP surface is written in a pairwise-additive way via individual nonbonded interaction potentials *U*_*i*_^*AA*–*NP*^corresponding to each *i*th CG AA bead. This potential depends on the distance *d*_*i*_ between COM of NP and *i*th AA. At the same time, the distance *d*_*i*_ depends on the orientation of the whole protein to the NP surface at the selected point *A*. This orientation is defined by two rotational angles θ and ϕ relative to the initial protein orientation defined in the PDB file (Figure 2). For the protein with *N*_*AA*_ residues, the protein–NP interaction potential at selected point *A* with selected protein orientation is written as
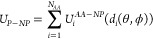


At the same time, the CG interaction energy for each AA at selected point *A* with selected protein orientation is the sum of nonbonded (van der Waals, dipolar, and excluded volume) and electrostatic terms:
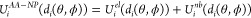


For the purpose of calculations of the electrostatic potential between NP and AA defined in terms of surface potential *φ*_*s*_, the distance *h*_*i*_ (*d*_*i*_*, θ, ϕ*) = *d*_*i*_ (*θ, ϕ*) – *R*_*NP*_ between the center of mass for AA and the surface of the NP is utilized instead of *d*_*i*_. In this form, the electrostatic potential implicitly accounts for properties of the environment, e.g., ionic strength *I* of the background electrolyte and the dielectric constant of solvent ε:
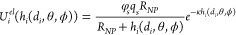
where κ = *λ*_D_^–1^ = (8π*l*_B_*Ι*)^1/2^ is the inverse Debye length, and *l*_B_ = *e*^2^/(4π*εε_0_k*_Β_*T*) is the Bjerrum length.

The nonbonded interaction potential *U*_*i*_^*nb*^ between *i*th AA and NP has two terms describing a short-range nonbonded interaction of each AA with the surface (*U*_*i*, *s*_^*nb*^) of NP and a long-range interaction with the core (*U*_*i*, *c*_^*nb*^) of NP. Both terms depend on the *h*_*i*_(*d*_*i*_, θ, ϕ) distance between the NP surface and the COM for AA:
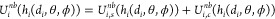


A short-range potential *U*_*i*, *s*_^*nb*^ is provided in tabulated form and precalculated by all-atoms MD simulations. The final summation of all terms described above for *N*_*AA*_ protein residues yields the collection of interaction energy values for a whole protein located at point *A* and rotated by angles (*θ*_*k*_*, ϕ*_*l*_). Repeating the same procedure of sampling energy as a function of rotational angles (*θ*_*k*_*, ϕ*_*l*_) at new positions along *z*-axis will produce a distribution of *U*_*P–NP*_(*z, θ*_*k*_*, ϕ*_*l*_) potentials corresponding to a multitude of states that an NP–protein adsorption complex can visit.

If the surface is represented by the flat slab, the mean interaction energy for a particular orientation of the protein (*θ*_*k*_*, ϕ*_*l*_) within a distance interval 0 ≤ *z* ≤ *a* can be evaluated as
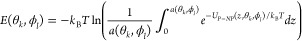


Plotting *E*(*θ*_*k*_, *ϕ*_*l*_) values in (*θ*_*k*_*, ϕ*_*l*_) coordinates results in the heatmap shown in Figure 2 which provides the information on adsorption energies in different orientations (*nanodocking*). Averaging *E*(*θ*_*k*_, *ϕ*_*l*_) values over all possible combinations of (*θ*_*k*_, *ϕ*_*l*_) with Boltzmann weighting factors *P_kl_* yields the final adsorption energy *E*_ads_, which can be used to rank different proteins by their adsorption affinity to selected material:
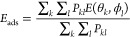

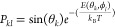


A *UnitedAtom* coarse-graining scheme of bio-nano interfacial interactions leads to a significant reduction of the number of degrees of freedom and cuts the computational cost. Adsorption energy heatmaps calculations facilitate the rapid detection of the most preferable orientation of the protein on the NP’s surface. However, it should be mentioned that the method provides the approximate solution to finding the preferred orientation and adsorption affinity rankings mostly due to the lack of flexibility of the protein and omitting entropic terms related to these degrees of freedom.^19^ For a higher precision calculation, a reverse mapping from the CG representation to the atomistic representation is needed, but until now, it is beyond the *UANanoDock* capabilities. For additional details, consult the subsequent section entitled Increasing the Resolution of the *UANanoDock* Predictions.

### I/O for the *UANanoDock*

To run the UANanoDock simulation, it is necessary to provide the following input:

- PDB coordinates of the protein. They can be obtained either from Protein Structure Data Bank^8,9^ or AlphaFold database^20^ or modeled directly from the sequence *ab initio* by I-TASSER^21^ or Rosetta software.^22^
- The pH of the solvent which surrounds the NP and the protein. The protonation state of the protein corresponding to the selected pH condition is modeled by the *PropKa*(23) routine included in *UANanoDock* preprocessing pipeline.
- The ionic strength of the solvent. The default value is 0.15Me^2^ which corresponds to the ionic strength of blood.
- The temperature of the simulated system in Kelvin.
- The material of the NP. Until now there are parameters for Ag, Au, Fe_2_O_3_, PEG, SiO_2_ amorphous, SiO_2_ quartz, TiO_2_ anatase, and TiO_2_ rutile. The list of materials will be increased in future updates.
- The NP’s radius in nm.
- The ζ, the electrostatic (or electrokinetic) potential of the material in mV.
- The *hkl* plane of the NP in the case of crystalline materials. For amorphous materials, the default *hkl* values are selected to be “000” by convention. There are no differences in the structure of different planes on amorphous materials.
- The desired angular resolution, by selecting angle step δ for sampling protein orientations and number of samples *N*_bin_ used for energy weighting per selected angular bin δ × δ.

The output of the *UANanoDock* model includes:

- A heatmap graph in PNG format is shown in Figures 2b which illustrates the average Boltzmann adsorption energy per orientation (in kJ/mol). Each orientation on this map is defined with ϕ and θ rotational angles using a step of two degrees and the corresponding energy term *E*(*θ*_*k*_, *ϕ*_*l*_).
- A UAM file, which contains the ϕ and θ angles used for the adsorption energy calculations. The ϕ and angle values start from 0° for both and reach 360° and 180° with selected angle step, respectively. The *propKa2UA.uam* file contains the adsorption energy per each ϕ and θ as well as the minimum surface distance of a protein’s bead from the surface of the NP. These values are used to make the heatmap mentioned previously.
- A DAT file (*material.dat*), which contains the information about the AAs-NP’s Hamaker constants that have been used for the selected NP’s material and plane.
- PDB and XYZ files of the preferred configuration of the protein immobilized on NP’s surface for further visualization and simulations.
- CONFIG file to be used with the desktop version of *UnitedAtom* software directly using the code which is available in ref (13).

### Web Interface for *UANanoDock* Software

**Figure 3:**
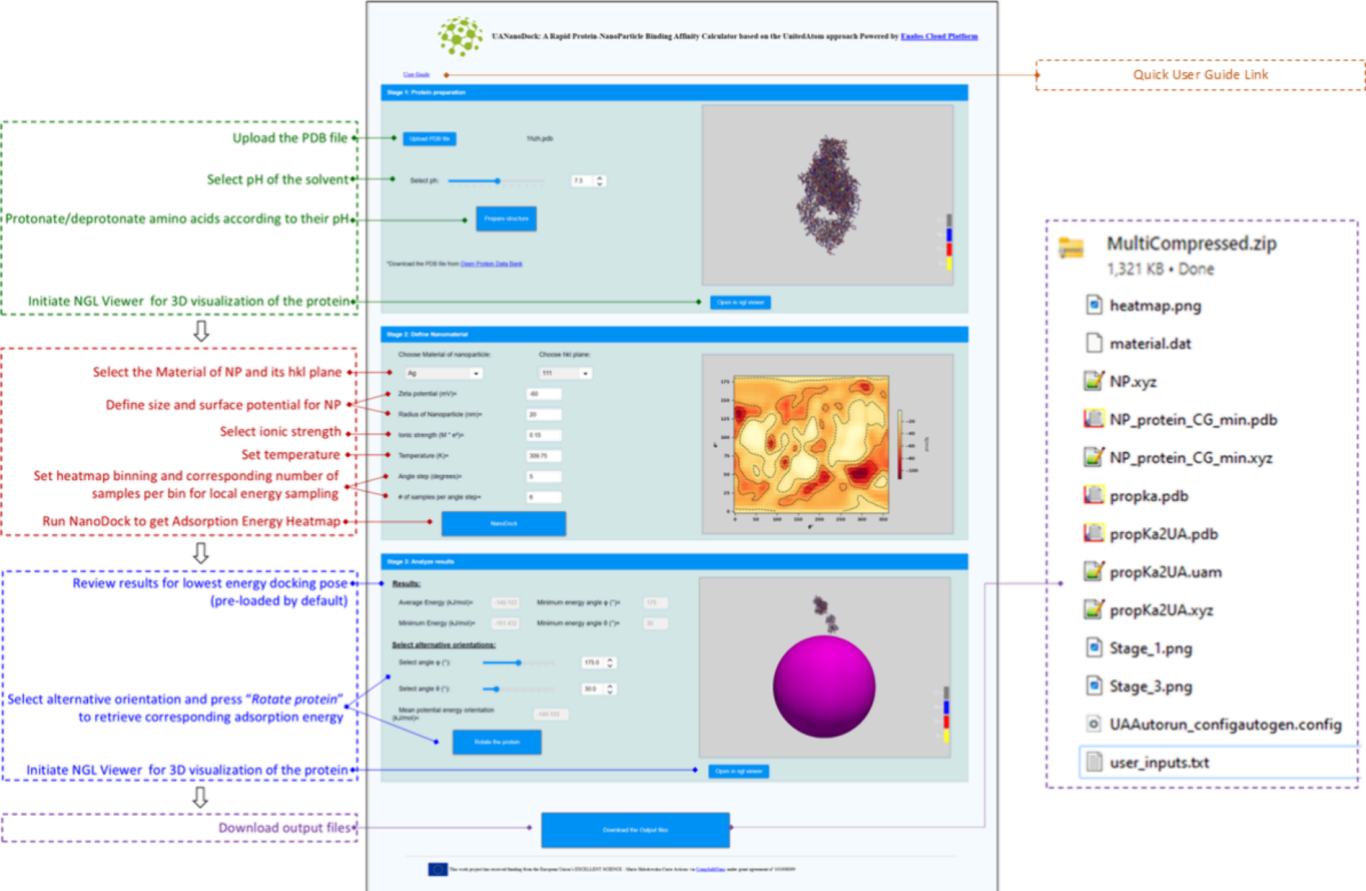
Graphical user interface of the *UANanoDock* web tool with a flowchart describing its steps.

The web interface of *UANanoDock* is divided into four different stages (see Figure 3). The first stage of *UANanoDock* includes uploading a PDB file of a protein and selecting the pH of the medium. After that, the “*Prepare Structure*” button can be clicked to set the protein AAs to their most favorable protonation state for the selected pH. Next, the user can create a visualization of the prepared protein structure by clicking on the “*Open in NGL Viewer*” button. The inclusion of various functionalities in NGL Viewer^24,25^ provides an extra benefit to users as publication-ready images can be created and downloaded.

In the second stage of *UANanoDock*, the properties of the NP (e.g., the material, surface potential, and radius) as well as the ionic strength and temperature of the surroundings are selected by the user. The user may optionally also choose the resolution at which the calculation should be performed, which we discuss in more detail later. Following this, the user clicks on the “*NanoDock*” button, which calls the *UnitedAtom* program with the selected parameters. The internal version of *UnitedAtom* implemented in the *UANanoDock* platform corresponds to release v1.0.4 of the *NPCoronaPredict* repository, with the *UA* version number of 1.1.0.^13^ Once this has finished running, a heatmap plot is produced and shown to the user in the imaging pane on the right. Following this, the user can select a specific protein orientation and click on “*Rotate the Protein*” button. Then, the user can see the adsorption energy of this orientation as well as its visualization through the NGL Viewer after clicking on the “*Open NGL Viewer*” button. During the fourth stage, the user can download the output files (heatmap, UAM file, DAT files, CONFIG files, etc.) for further analysis.

By default, during the operation of *UANanoDock*, the angular resolution, i.e., the number of different protein orientations sampled, is set to a step size of δ = 5°, with *N*_bin_ = 6 samples of random orientation used for energy sampling within each 5° × 5° bin. This is done as a trade-off between generating a high-resolution heatmap and acquiring adequate sampling statistics in each bin without requiring computational time in excess of the available resources. The user is given the option to change these parameters subject to a cap on the total number of calculations performed (*N*_total_ = 16200) per protein–NP system, that is, (360°/δ) × (180°/δ) × *N*_bin_ < *N*_total_. This limitation has been added to ensure the stability of the web application. Based on preliminary tests, the locations of the minima on the heatmap are predominantly independent of the selected resolution; yet, their relative depths exhibit slight shifts, resulting in a displacement of the absolute minima. It is important to note that irrespective of the selected resolution, an inherent uncertainty remains associated with the predicted relative adsorption energy for these minima. This arises from limitations of the underlying model, the uncertainty in the supplied PDB files (typically accurate to a resolution of 1 Å), and the rigid-protein approximation. Consequently, the predicted lowest energy docking pose may not necessarily correspond to the experimentally observed configuration, and the use of a higher precision in terms of the angular resolution does not produce more physically meaningful results. Thus, it is more appropriate to consider multiple local minima. Due to this computational uncertainty, all energies within ±1 standard deviation of the absolute minimum energy value (*SD(E*_ads_*)*) should be considered as potential docking poses. The standard deviation values for each orientation are written to the UAM file in the “SDEV(Eads)” column). For example, for IgG-AgNP (*R* = 15.0 nm, ζ = −20.0 mV) adsorption discussed in the section below, the absolute minimum was located at [ϕ,θ] = [180°,30°] and had *E*_ads_ = −185.91 kJ/mol with *SD(E*_ads_*)* = 4.45 kJ/mol. Therefore, all local minima below −181.46 kJ/mol warrant consideration. In general, one cannot strictly anticipate Boltzmann equilibrium for protein adsorption on realistic time scales due to the strong adsorption energies resulting in extremely slow desorption rates. This is particularly true for strong-binding metallic NPs interfaces, where equilibration would require a duration on the order of years. This suggests that many local minima are likely to be observed, and it would be erroneous to assume that the lowest energy orientation would correspond to that observed in an experimental one. It is strongly recommended that the obtained docking poses be treated as low-resolution models for protein–NP complexes, and similarly to small-molecule docking methodology, they should undergo further refinement through more sophisticated molecular dynamic modeling to obtain more precise configurations of the adsorption complex.

**Figure 4:**
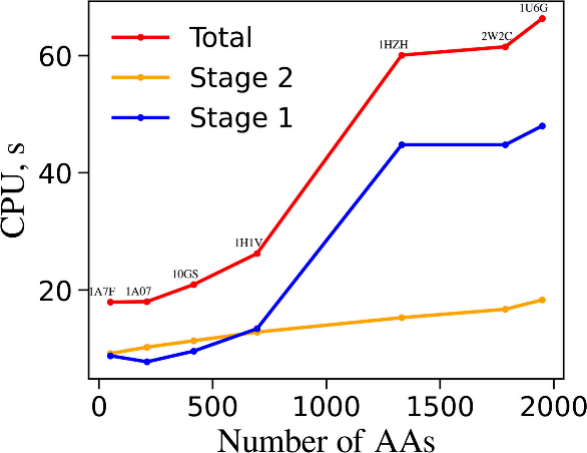
*UANanoDock’s* CPU time in seconds for Stage 1 (blue line), Stage 2 (orange line), and both stages (red line) as a function of the number of AAs in the protein. The protein RCSB names used appear above the blue points, while their digital object identifiers (DOI) are mentioned in Table S1 of the Supporting Information. The angular resolution selected for these calculations was δ = 2° with bin sampling *N*_bin_ = 1.

Performance benchmarking of *UANanoDock* as a function of protein length is shown in Figure 4. The first stage of protonation/deprotonation of the protein is longer than the next step of *UnitedAtom* calculations in the case of proteins with more than 1000 residues. This is the slowest stage of the *UANanoDock* tool. Stage 2 appears to have a linear dependency on the length of the protein sequence: *y* = 0.0045*x* + 9.3224, where *y* is the CPU time, *x* is the number of AAs, and corresponding Pearson correlation coefficient *R*^*2*^ = 0.9893, in agreement with the theoretical expectation that the program is linear with respect to the number of residues due to containing a loop over these. The total CPU time for the longest protein with approximately 2000 residues was less than 70 s. This CPU performance allows *UANanoDock* to be used as a web application.

### Quick User Guide and Tutorial for *UANanoDock* Software

The instructions on the use of the *UANanoDock* tool are available in the form of a quick user guide and extended Python Jupyter Notebooks tutorial. Access to the first one is provided via the “User Guide” link on the Enalos platform (Figure 3, top left). The extended tutorial, which repeats the steps of the quick user guide and shows the example of reusing files generated with the Enalos platform for more detailed *UnitedAtom* simulations, analysis, and NGLView visualization on the personal desktop, is available via GitHub (https://github.com/juliasubbotina/uananodock_tutorial).

### Increasing the Resolution of the *UANanoDock* Predictions

The accuracy of the *UANanoDock* predictions can be compromised by various elements. A crucial concern is the quality of protein structure coordinate information, which may be impacted by either imprecise models derived from experimental studies (such as those found in the Protein Structure Data Bank) or by estimations generated through computational approaches (like those in the AlphaFold database). To address this issue, it is recommended that structure-refining algorithms be employed prior to *UANanoDock* computations. A series of steps should be invoked prior to docking studies: verifying the physiologically relevant protein composition (e.g., determining whether it exists as a monomer or multimer), adding missing AA residues, and pre-equilibrating the structures. In previous research, we demonstrated that the predicted binding energies of 59 blood serum proteins onto spherical TiO_2_ and Au NPs with radii ranging from 5 to 200 nm, utilizing both experimentally and computationally derived structures, exhibited a ±20% relative error between the two sets of protein coordinates.^33^ The process of docking disordered proteins poses unique challenges, particularly in establishing suitable initial coordinates. While pre-equilibration of these proteins can enhance predictive accuracy, it introduces significant computational demands. Our investigations into caseins adsorption onto metallic NPs,^34,35^ a class of predominantly intrinsically disordered milk proteins, have revealed that achieving a stable root-mean-square deviation (RMSD) of 0.2–0.7 nm necessitates approximately 20 ns of computational time. Furthermore, the “rigid body” approximation implemented in the UA model does not account for potential protein unfolding during interactions with nanomaterials.^19^ Although the structure of globular proteins appears to remain predominantly preserved as they maintain their biological functions, the impact of unfolding on binding energies should not be disregarded. It can be reasonably inferred that increased protein flexibility, whether intrinsic or induced, correlates positively with greater uncertainty in nanodocking predictions. Adding a post-docking refinement step with additional MD simulations for a set of docking poses may provide more insights into relative stability of predicted adsorption complexes. In this regard, nanodocking is similar to the docking of small molecules to receptor proteins: the more degrees of freedom that can be introduced in the calculations, the better the result. The incorporation of additional MD routines into the *UANanoDock* web tool presents a significant computational challenge, necessitating a careful balance between enhanced functionality and resource allocation.

Another source of uncertainty in docking predictions is associated with the complexities involved in developing atomistic FF for materials (for instance, the decision to include or exclude polarization).^2^ It is essential to acknowledge that *UnitedAtom/UANanoDock* may also be susceptible to these FF-related inherent limitations due to its reliance on all-atom simulations for the parametrization of surface potentials *U*_*i*, *s*_^*nb*^. As the field of FF development in materials science advances, it is anticipated that the *UnitedAtom/UANanoDock* model can be refined to improve the description of AA-materials interactions.

The *UANanoDock* online platform was engineered to assist individuals who find it challenging to utilize the desktop version of *UnitedAtom/NPCoronaPredict,* as the latter was primarily constructed for executing corona prediction simulations rather than examining single protein interactions and alignments. As users gain proficiency with the *UnitedAtom* methodology via the web-based tool, they are presented with the opportunity to acquire all of the essential files for conducting custom *UnitedAtom* simulations with expanded capabilities on their machines. After downloading the archive with online results, users have the flexibility to incorporate their custom set of surface potentials *U*_*i*, *s*_^*nb*^ (e.g., precalculated with a different FF) into *UnitedAtom* by exchanging the original PMF files in the corresponding directory and implementing minor modifications to the CONFIG files. Instructions for this process were provided in the GitHub tutorial. The implementation of this feature may serve to address inaccuracies stemming from the limited capacity of the atomistic FF to accurately represent bio-nano interfacial phenomena.

The terminology and interpretation of the *UANanoDock* predictions depend on the relative size of the NP and the protein. While for large NPs or diameters greater than 10 nm one can discuss the protein–NP complexes in terms of NP protein corona and expect multiple proteins adsorbed on a single NP, for smaller NPs we may take another perspective and talk about multivalent binding and bioconjugation to address the situations where one or more NPs are bound to the same protein molecule. In the case of small NPs, the tool can be used to find the preferred pockets for the NP binding on the protein surface. We note that both scenarios can be addressed with the *UANanoDock* tool, although only two entities can be modeled in each step. A limitation of the method is the rigid-molecule approximation, due to which the binding energy can be underestimated and some preferable configurations missed.

### The *UANanoDock* Case Study: Rational Design of Immunoassays Based on IgG1 Antibody

**Figure 5:**
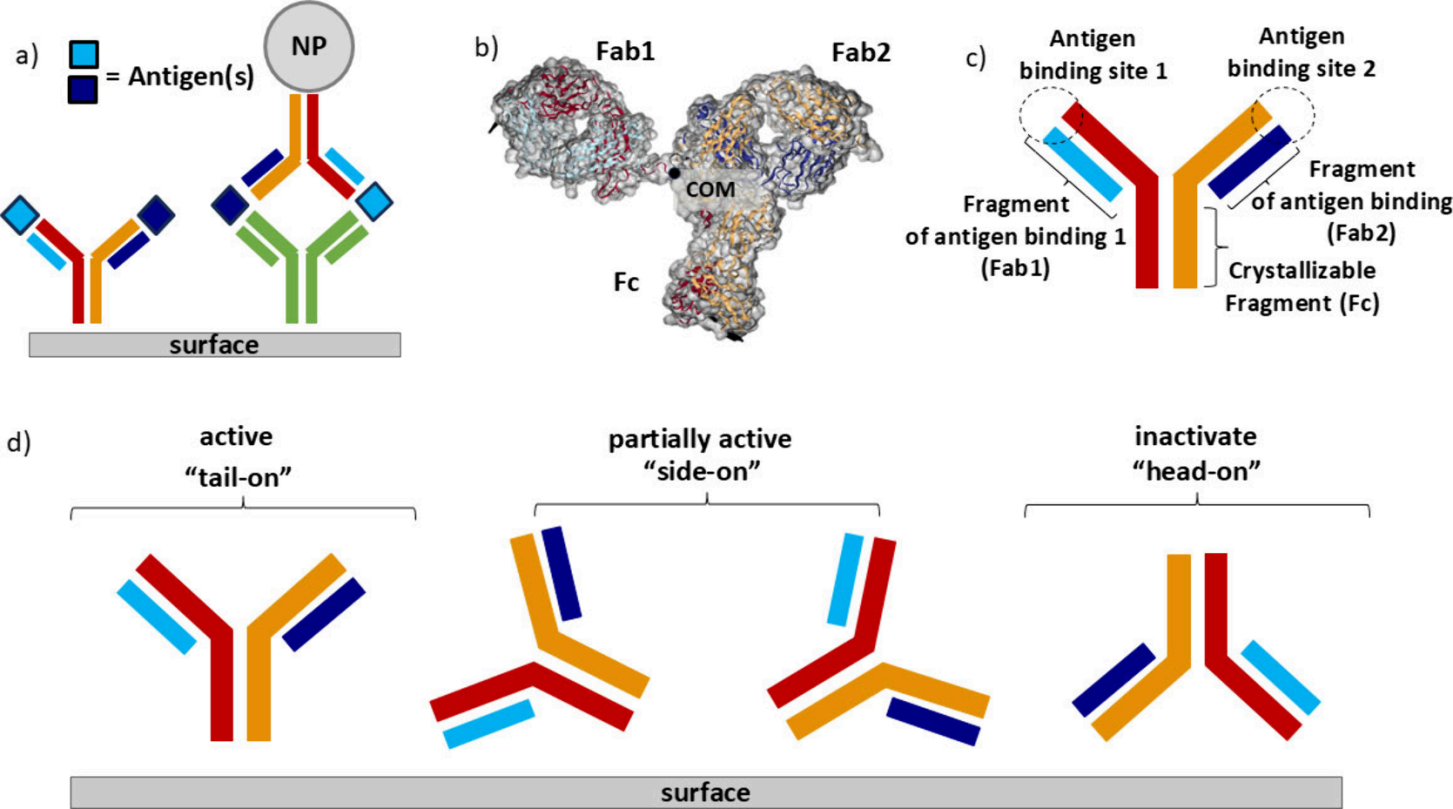
(a) Working principle for IgG-based immunoassays. (Left) Direct detection of antigens by capturing antibodies immobilized on a solid substrate. (Right) Detection of antibody-captured antigens by analytical antibody–NP conjugates (NP-enhanced LFIA technique). (b) IgG antibody with its principal axes and its center of mass (COM). Four polypeptide chains appear in red, yellow, light blue, and dark blue. *UANanoDock* assigns the axis with the highest moment of inertia to the *z*-axis by convention. (b) Sketch of the IgG antibody and its four polypeptide chains. Each color corresponds to the polypeptide chains mentioned in (c). The single crystalline fragment (Fc) and two antigen-binding sites Fab1 and Fab2 are defined as shown. (d) Possible orientations of immobilized IgG antibody at the surface of NPs. (Left) Antibody orientation has its single crystalline fragment (Fc) attached to the NP surface and having its antigen binding sites free for interactions. This is a desired spatial arrangement. (Middle) Antibody orientation has one of its antigen binding fragments (Fab 2) attached on the NP surface and its Fc fragment partially attached. This is a partially active “side-on” orientation and is less capable of antigen detection. (Right) Antibody orientation having both of its antigen binding sites partially attached on the NP having Fc fragment free to be attached into the immune system cells surface, treating NP as a pathogen (if the NP formulation is administered intravenously). This orientation is unsuitable for antigen detection.

Traditional IgG-based immunoassays typically have specific antibodies directly bound to the solid surface (Figure 5a, left). The attachment to the solid surface can be covalent or achieved by physical adsorption. Noncovalent immobilization is preferred, as it is a simple and cost-effective technique driven by electrostatic, ionic, hydrophobic, hydrophilic, and van der Waals forces.^26^ Nevertheless, due to the inherently weaker nature of these forces compared to covalent bonds, bioassays utilizing this attachment method may be less specific. While noncovalent antibody attachment is universally applicable, it often leads to random protein orientation on the surface, diminishing the assay’s effectiveness in antigen detection. To enhance the selectivity of immunoassays, NPs can be integrated into their design (Figure 5a, right). One of the designs includes a NP that carries antibodies (e.g., IgG, see Figure 5b)^17^ attached in a specific orientation (via Fc domain) where both antigen-binding domains (Fab1 and Fab2) remain free for capturing antigens floating in the sample (Figure 5d, left).^27^ The noble metal NPs were specifically useful in lateral flow immunoassays (LFIA) where they have been shown to amplify the selectivity.^28^ Controlling the orientation of immobilized antibodies can be achieved by altering surface charges on either the solid substrate or the protein itself. For the former, this involves modifying coatings and surface chemistry^29^ on NPs. For the latter, changes can be made to the protein’s charge distribution by adjusting the protonation state through pH manipulation or by performing site mutagenesis in the antibody sequence.^30,31^

Conducting laboratory experiments to test various immunoassay parameters and conditions for their development is a costly endeavor. Computational methods, such as MD simulations, can potentially help to narrow down the optimal range of conditions for protein immobilization.^2^ However, the complexity of these macromolecular systems also limits the applicability of MD simulations for virtual prescreening of numerous immunoassay designs. From this perspective, the *UnitedAtom* method is extremely suitable for computational prescreening of the different nanomaterials in different shapes, sizes, and surface chemistry and different protein sequences existing at different protonation states.^10−12,19,32−37^

We applied the *UANanoDock* web tool to identify optimal conditions of the IgG immunoglobulin (UniProt ID: P01834) immobilization onto the surface of noble metals, specifically silver NPs (AgNPs). The ranges of the NP radius (i.e., 2, 4, 8, 10, 15, 20, 25, 30, 35, 40, 45, and 50 nm) and NPs surface potentials (i.e., 0, −10, −20, −30, −40, −50, −60 mV) were tested. The uncoated AgNP’s isoelectric point occurs at a low pH and is determined by the material itself, not the NP size. To simulate more realistic systems, nonpositive surface potentials have been selected. The NP size influences the surface potential magnitude, which is also affected by solvent parameters like ionic strength and dissolved ion types.^38−42^ To better represent the electrostatic interactions between the NP and IgG antibody, a range of surface potentials was examined. It is worth noting that the *UnitedAtom* method cannot directly model NP aggregation following occasional pH changes; instead, this was addressed by varying the NP radii. The AgNPs’ typical X-ray diffraction pattern shows the highest intensity peak corresponding to Miller indices *hkl* = 111^43^ corresponding to the face-centered cubic symmetry of the material. Therefore, we used 111 Ag surfaces for our study.

**Figure 6:**
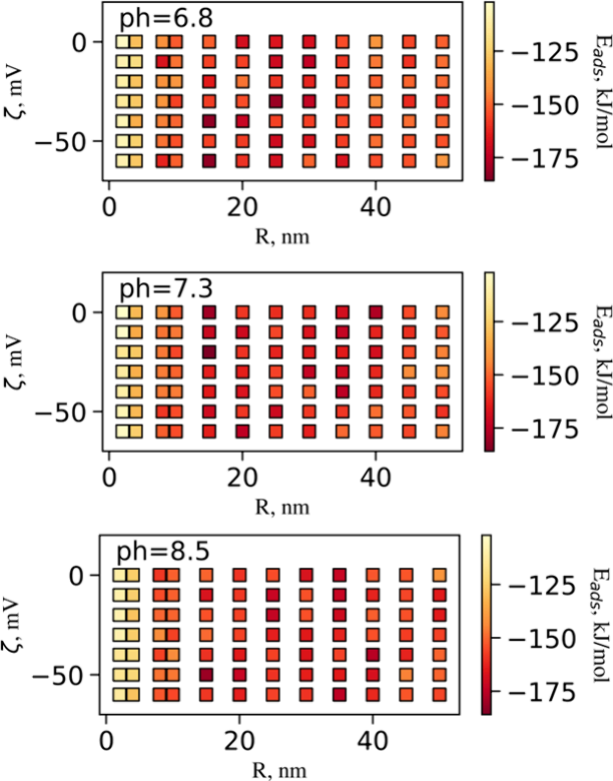
Identifying the optimal conditions for protein binding. The NP radii were in the range between 2 and 50 nm. For this graph, the absolute minimum energy values were considered. The markers are filled with color, as per the energy bar shown on the right. The binding of IgG to the NP surface was exothermic under all conditions (*E*_ads_ < 0). Almost identical adsorption energy levels were predicted for AgNPs with a radius *R* = 15 nm with different surface potentials. Lower adsorption affinity was predicted for smaller NPs with *R* < 15 nm. However, the relationship between adsorption energy and the charge regulation factors, surface potential, and protein charge corresponding to the protonation state at a given pH value was inconsistent. The lowest *E*_ads_ was predicted for IgG adsorption at pH 7.3 onto AgNP with *R* = 15.0 nm and ζ = −20.0 mV. The angular resolution selected for these calculations was δ = 5° with bin sampling *N*_bin_ = 6.

**Figure 7:**
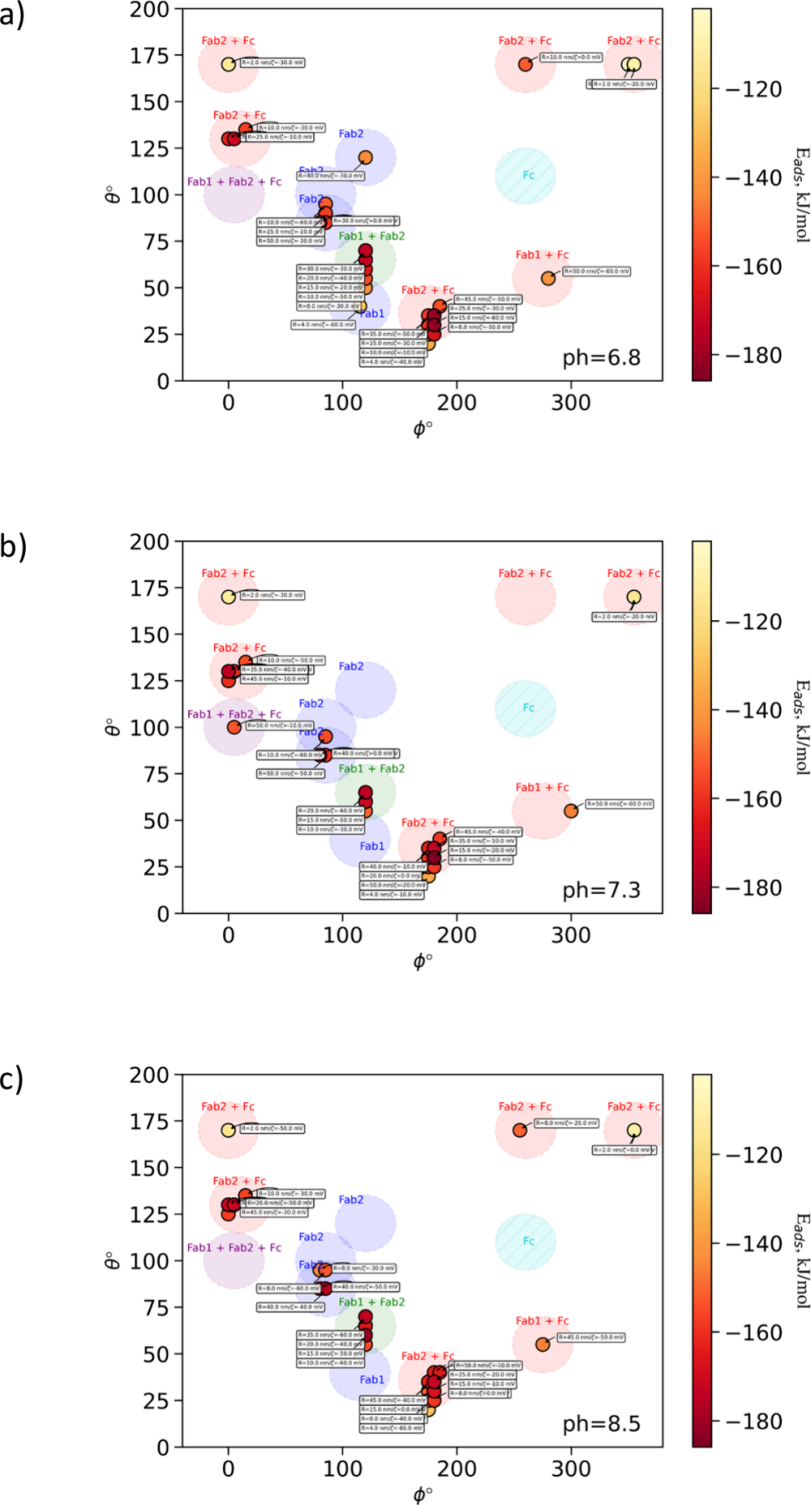
*UANanoDock-*predicted protein binding orientations at different conditions (*R*, pH, and ζ). Only the lowest energy conformations from individual heatmaps are included. The colors of the markers correspond to the strength of the adsorption energy for the lowest docking pose shown in the bar on the right. The colored patches on the plot mark locations of predicted orientations: (blue) Fab1 or Fab 2 are in contact with the NP (partially active “side-on”), (red) Fc and one of the antigen binding domains Fab1 or Fab2 are in contact with the NP (partially active “side-on”), (green) both antigen-binding domains Fab1 and Fab2 are in contact with the NP (inactive “head-on”), (purple) all fragments Fab1, Fab2, and Fc are in contact with the surface (inactive “flat-laying” conformation). None of the predicted orientations could be assigned to the fully active “tail-on” orientation marked with a turquoise patch at [ϕ,θ] = [260°,110°], when IgG is bound via the Fc fragment and both Fab fragments are facing outward and are thus available to capture antigens.

**Figure 8:**
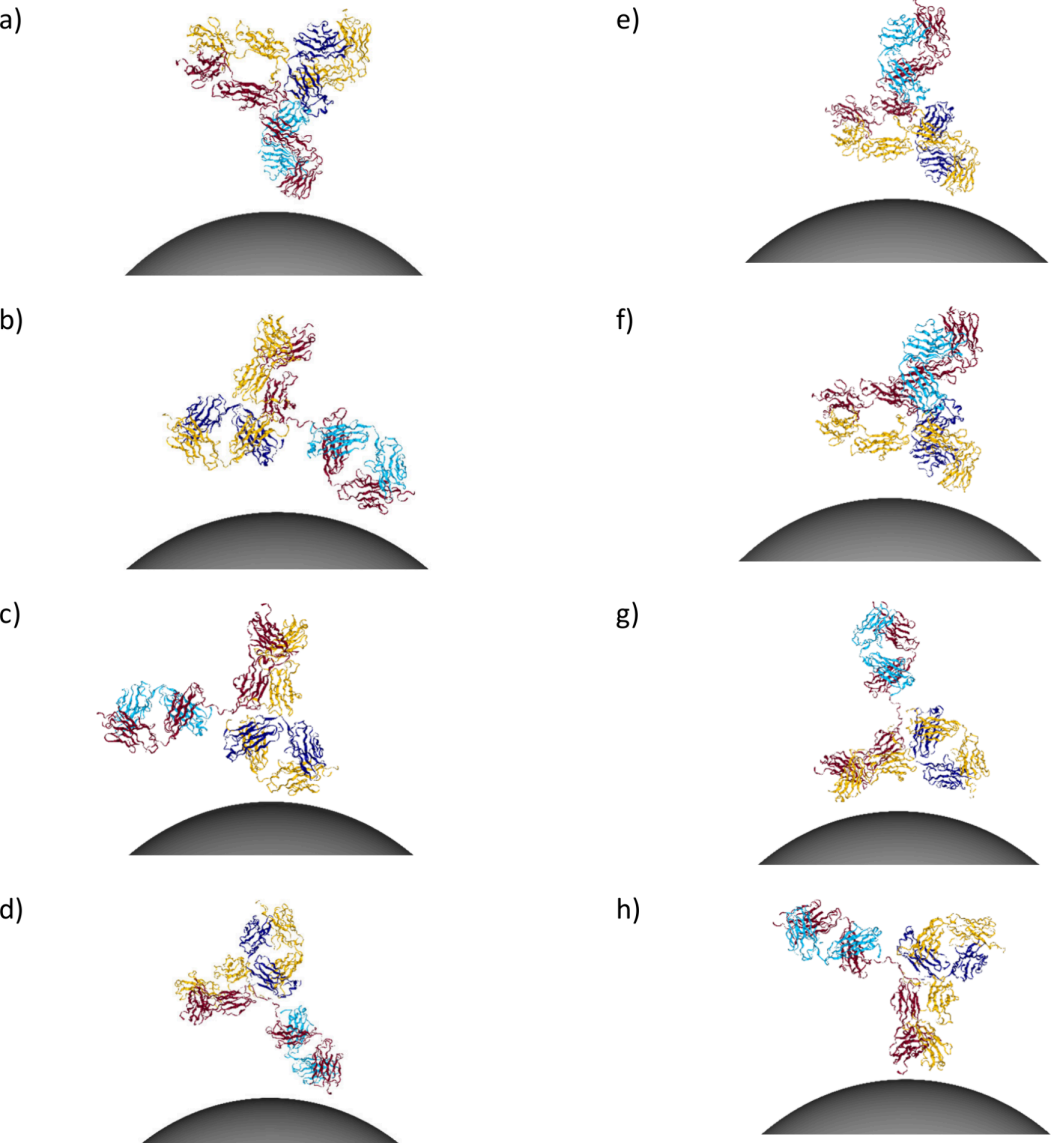
*UANanoDock* predicted protein–NP conjugates. The shown orientations of IgG adsorbed on 15 nm AgNP (*hkl* = 111, ζ = −20 mV, pH = 7.3) correspond to the seven lowest energy docking poses located at the adsorption heatmap (see Figure 9). The fragments closest to the NP surface were (a) Fab2 and Fc, [ϕ,θ] = [180°,30°], (b) Fab1 and Fab2, [ϕ,θ] = [120°,60°], (c) Fab2, [ϕ,θ] = [85°,90°], (d) Fab1, [ϕ,θ] = [215°,40°], (e) Fab2 and Fc, [ϕ,θ] = [20°,130°], (f) Fab1 and Fc, [ϕ,θ] = [280°,45°], (g) Fab2 and Fc, [ϕ,θ] = [300°,165°]. Orientation b had the lowest energy value and was included in Figure 7. Orientations b–f were above the +1SD cutoff from the absolute energy minimum. Orientations e–g are “side-on” complexes with very similar spatial arrangements. Orientation h is the desired “tail-on” orientation, [ϕ,θ] = [260°,110°]; however, it was not predicted to occur.

The coordinates of the IgG antibody were taken from PDB.org (PDBID: 1HZH). The temperature of 309.75 K, the ionic strength of 0.15M*e*^2^, and the set of pH values of 6.8, 7.3, and 8.5 were used to imitate slightly acidic, neutral (corresponding to human blood pH^44^), and slightly basic conditions for protein adsorption, respectively. The *PropKa* methodology implemented into *UANanoDock*(13,23) was applied to identify the correct protonation state for AAs side chains at selected pH. Furthermore, any nonstandard entities (e.g., cocrystallized molecules or post-translational modifications) were removed from the PDB file automatically by preparations at *UANanoDock*’s Stage 1. The PDB file derived from Stage 1 was used as an input to the docking procedure. To scale up this *in silico* prescreening of adsorption conditions, the input files obtained from the *UANanoDock* web tool were rerun locally with the most recent *UnitedAtom* version (1.2.0) to obtain the data that are discussed below. The angular resolution of δ = 5° with *N*_bin_ = 6 was used for all runs (local and online). The results of these calculations are shown in Figures 6–8. The adsorption energy, the binding site, and the preferential rotational angles of the IgG antibody onto NP’s surface have been calculated and are provided in the Supporting Information .txt file.

Analysis of the obtained data suggests that the radii of the NPs play a stronger role in IgG adsorption affinities than their ζ-potential. However, this dependency is not linear: The interactions between Ag surface with Miller indices *hkl* = 111 and IgG were the strongest at intermediate values of NP’s radius *R* = 15 nm (see Figure 6). These results echo the observation for gold NPs, where smaller particles collectively absorbed more IgG than the larger ones.^45^ However, very small AgNPs have shown less binding capacity as compared to NP sizes of *R* > 15 nm. The surface charges did not affect adsorption affinities significantly. In general, the AgNPs were found to have a strong overall affinity for IgG, as predicted average adsorption energies were negative for any selected combination of parameters.

**Figure 9:**
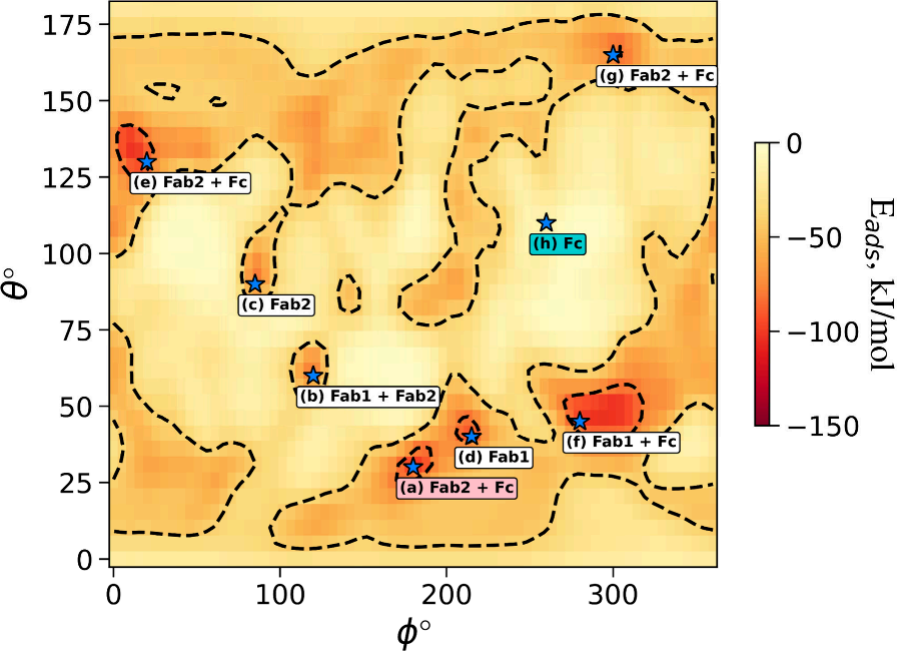
*UANanoDock* adsorption heatmap (in kJ/mol) for IgG-AgNP (*hkl* = 111, R = 20 nm, ζ = −60 mV, pH 7.3). Locations of protein–NP conjugates shown in Figure 8 are marked with the “star” sign. One should notice that multiple binding orientations are possible (some of them are similar; see Figure 8) and can be used as a starting point for further simulations to design system modifications with increased binding affinities. The label for the lowest-energy orientation (a) is annotated in pink. No local minimum is seen at [ϕ,θ] = [260°,110°] for the desirable “tail-on” orientation (h), whose label is highlighted in turquoise. The angular resolution selected for these calculations was δ = 5° with bin sampling *N*_bin_ = 6.

Considering the dipole direction on IgG,^46^ the expected orientation of the protein at neutral pH on the surface of negatively charged AgNPs should be either “head-on” (both Fab fragments facing the nanomaterial) or “side-on” (one of the Fab fragments and Fc subunit facing the surface). Analysis of lowest energy conformations from the obtained heatmaps confirms this prediction: no “tail-on” oriented adsorption should occur. Instead, random “side-on” and “head-on” adsorption are found (see Figures 7 and 8, also see Figure 9). The inactivated “head-on” configurations with both Fab fragments facing the NP surface and partially active “side-on” orientations with Fab2 and Fc facing the NP were calculated to be the most stable. The Fab2 segment appears to function as the primary binding site for sustaining the connection with a particular set of AgNPs, while the Fab1 and Fc segments seem to provide additional stabilization. The anticipated “tail-on” configuration is predicted to be exothermic, although it is not the most favorable arrangement, and no local minima were associated with it at the adsorption energy landscape. Based on these predictions, the system of IgG conjugated to simple uncoated AgNPs investigated in this case might be less than optimal for LFIA applications. Further design modifications, such as the replacement of IgG with its mutant form or coating of AgNPs, may potentially shift the adsorption tendencies toward the desired orientation. For users seeking to scale up and to develop more advanced and efficient *in silico* workflows for rational design of nanobio platforms, it is recommended to utilize the desktop version of *UnitedAtom* software.^13^

## Conclusions

The *UANanoDock* web tool for the investigation of protein adsorption onto NP surfaces has been developed and made freely available for the needs of the research community working on the development of various nanoplatforms. It applies the *UnitedAtom* approach to calculate the adsorption affinities of the protein for different NPs and predicts the most likely orientations of the immobilized protein. The protein adsorption energy heatmap is also calculated and encodes the information on the energy landscape for noncovalent interaction between protein and NP in terms of rotational coordinates ϕ and θ. Modeling of charge regulation of protein adsorption in response to the change of pH or ionic strength of solution is also implemented. Upcoming iterations of *UANanoDock* will expand its modeling capabilities by incorporating a broader range of materials. This includes an extended database of polymer-based substances such as polystyrene, polyethylene terephthalate, polypropylene, and polyvinyl chloride. Additionally, there are plans to address the rigid-protein constraint by implementing new custom features. These features will adjust PMFs based on the *B*-factor values stored in the PDB structure.

Due to the high performance (e.g., processing a 1331 AA protein in less than a minute) and user-friendly design, *UANanoDock* can be utilized by users without a programming background and with no need to install the software locally. An example of *UANanoDock* use for the rational design of theranostic nanodevices, e.g., immunoassays, was also discussed in the current communication. We have shown that IgG attachment onto metallic AgNP surfaces occurs mostly via antigen-binding site Fab2, in some cases jointly with Fab1 or Fc units. These results suggest that pristine AgNPs might require further functionalization to achieve satisfactory performance of IgG-based immunoassays based on NP binding of proteins and subsequent protein–antigen binding. To be consistent with the FAIR principles, a Modeling Data documentation (MODA) has been made available using the Easy-MODA^47^ tool and registry and is provided in the Supporting Information.

## Data Availability

Access to *UANanoDock* web tool is provided on a free basis via Enalos Platform (https://www.enaloscloud.novamechanics.com/compsafenano/uananodock/). The data discussed in this manuscript, including Python scripts used for data analysis and visualization, can be found on GitHub (https://github.com/juliasubbotina/acs_uananodock_data/). The MODA document has also been uploaded to the EasyMODA database of MODAs to enhance the FAIRness (Findability, Accessibility, Interoperability and Reusability) of models and software (https://www.enaloscloud.novamechanics.com/insight/moda, ID# *QdHvn*).

## Supporting Information Available

Additional tables and note (PDF)
Calculated docking poses (TXT)
Modeling Data documentation (MODA) (PDF)
