# Supplementary material for: UANanoDock: A Web-Based UnitedAtom Multiscale Nanodocking Tool for Predicting Protein Adsorption onto Nanoparticles
Source: J Chem Inf Model. 2025 Mar 25;65(7):3142–53. doi: 10.1021/acs.jcim.4c02292 (PMC12004535; doi:10.1021/acs.jcim.4c02292)
Supplement: Supplementary file 1 — ci4c02292_si_001.pdf [file ci4c02292_si_001.pdf]

Electronic Supporting Information:

UANanoDock: A web-based United Atom multiscale nano-docking tool for predicting protein adsorption onto nanoparticles

Julia Subbotina,[*,1] Panagiotis D. Kolokathis,[2,3] Andreas Tsoumanis,[3,4] Nikolaos K. Sidiropoulos,[2,3] Ian Rouse,[1] Iseult Lynch[5], Vladimir Lobaskin,[1] Antreas Afantitis[†,3,4]

[1] *School of Physics, University College Dublin, Dublin, Ireland*

[2] *NovaMechanics MIKE, Piraeus, Greece*

[3] *Entelos Institute, Larnaca, Cyprus*

[4] *NovaMechanics Ltd., Nicosia, Cyprus*

[5] *School of Geography, Earth and Environmental Sciences, University of Birmingham, Birmingham, UK*

[*]E-mail: yulia.subbotina@ucd.ie
[†]E-mail: afantitis@novamechanics.com

Table S1: Protein information (digital object identifiers (DOI), number of amino acids) and their central processing unit (CPU) time demands in *UANanoDock* Stages 1 and 2

PDB DOI	protein name	number of amino acids	CPU time (s) for *UnitedAtom* Stage	CPU time (s) for *propKa* Stage	total CPU time(s)
https://doi.org/10.2210/pdb1U6G/pdb	1u6g	1949	18.32	48	66.32
https://doi.org/10.2210/pdb2W2C/pdb	2w2c	1787	16.72	44.78	61.5
https://doi.org/10.2210/pdb1HZH/pdb	1hzh	1331	15.29	44.78	60.07
https://doi.org/10.2210/pdb1H1V/pdb	1h1v	695	12.83	13.4	26.23
https://doi.org/10.2210/pdb10GS/pdb	10gs	416	11.35	9.58	20.93
https://doi.org/10.2210/pdb1A07/pdb	1a07	211	10.26	7.76	18.02
https://doi.org/10.2210/pdb1A7F/pdb	1a7f	50	9.17	8.79	17.96

Note S1: On methodology of obtaining short range CG potentials with all-atom resolution of bio nano interface.

In short, a one-directional distance-dependent CG potential energy function *U* for two interacting particles can be described via probability distribution *P*, which represents the likelihood of observing specific values of the dependent variable corresponding to given values of the independent variable:

$$U = f(P),$$

Similarly, the radial distribution function *g(**r**)* can be interpreted as a probability density function, representing the chance of observing two particles separated by a particular distance **r**, e.g. the distance between centres of mass (COM) for two fragments composed of multiple atoms. The radial distribution function *g(**r**)* can be calculated from all-atom MD simulation trajectories [1]. Then, for the two- particle CG system the potential mean function (PMF) can be calculated as:

$$U(\mathbf{r}) = -k_B T \ln g(\mathbf{r}).$$

However, sometimes this approach might be insufficient in case of relatively large systems and requires to run long unbiased simulations to ensure that the modelled system would visit all possible states and proper probability distribution is sampled. To improve the probability sampling one can introduce bias, that forces the system to visit every state in a coordinate space of ***{r}***, e.g. umbrella sampling [2] or adaptive well-tempered metadynamics [3]. The unbiased probability distribution *P* an be obtained from biased ensemble by re- weighting and the PMF profiles then can be recovered by applying weighted histogram analysis method (WHAM) implemented in the *gmx wham* tool in Gromacs [4] if umbrella sampling scheme was invoked, or by PLUMED plug-in to Gromacs in case of adaptive well-tempered metadynamics (AWT-MetaD) [5]. These PMFs in tabulated form then can be used to describe a short-range potential near the NP surfaces (see $U_{i,s}^{nb}$, a non-bonding surface potential describing interaction between *i-th* individual amino acid side-chain in the protein and NP surface) where a high-resolution representation of the bio-nano interface is needed.

We use both approaches in parameterizing $U_{i,s}^{nb}$ potentials: for solid material we applied the AWT-MetaD, while for flexible polymeric chains we obtained potentials through inversion of RDFs, *g(**r**)*. The typical simulation boxes are shown in Figures S1 and S2. The simulation system typically includes fragments representing amino acid side chains and nanomaterial, e.g. slab (Figure S1) or *n*-meric chain of polymer (Figure S2). They are solvated and neutralised by adding ions.

Potentials for crystalline material are calculated as a function of surface-surface distance (d_{SSD}) between two surfaces of NP and *i-th* AA. The relationship between d_{SSD} and $h_i(d_i)/d_i$ as follows:

$$d_{SSD} = d_i - R_{AA} - R_{NP} = h_i(d_i) - R_{AA}$$

The *UA* CONFIG file contains information on R_{AA} and R_{NP} , thus the script can redefine PMFs as a function of d_{SSD} to PMFs as a function of $h_i(d_i)$ or d_i where needed.

Potentials for polymeric beads calculated from RDF functions that are calculated as function of d_i and should also be corrected with respect to the dimensions of the polymeric unit used for parameterization. These dimensions can be obtained from the radius of gyration.
When the unit dimensions fall significantly below 1.0 nm (which represents a typical x/y dimension of the simulation cell), it becomes necessary to implement additional corrections to ensure the PMFs can be appropriately scaled by *UnitedAtom*, which is typically the case for PMFs representing small polymer subunits e.g. trimers. In this case, an artificial "slab" of the material is constructed at a target density from these sub-units. Bulk PMFs are generated by summation of the component PMFs over sub-units in an artificial slab.

The detailed description of each type of simulation for parameterizing $U_{i,s}^{nb}$ potentials were previously described in multiple detailed reports. References to these publications are collected in Table S2 below.

Table S2: Information on protocol of obtaining shot range potentials for the materials available in *UANanoDock* parameters set.

Material	Method	Force Field	Protocol published in
Ag	AWT-MetaD	Interface FF [6]	[10]
Au	AWT-MetaD	Interface FF [6]	[11]
Carbon Black	AWT-MetaD	Custom FF [7]	[7]
Fe2O3	AWT-MetaD	Custom FF [7]	[7]
SiO2	AWT-MetaD	Custom FF [7]	[7]
TiO2	AWT-MetaD	Custom FF [8]	[8]
PEG	MD/RDF/PMF	CHARMM36 FF [9]	[11]

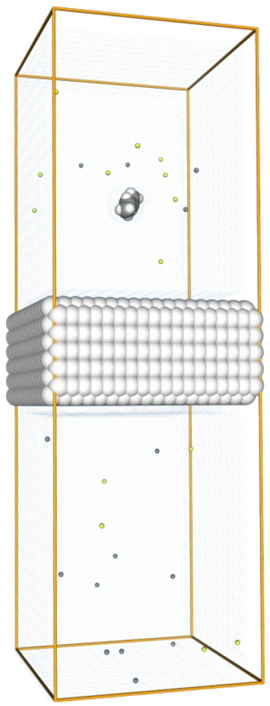

Figure S1. The example of a simulation box for parameterizing crystalline material – SCA interaction. The box of metallic gold interacting with the amino acid side chain is shown. The metallic slab has an approximate size of 3.5 nm by 3.5 nm by 2.5 nm. The amino acid side chain is placed near the surface. The whole setup is solvated with TIP3P water and neutralised by 0.15M KCL. The total height of the box is approximately 12 nm.

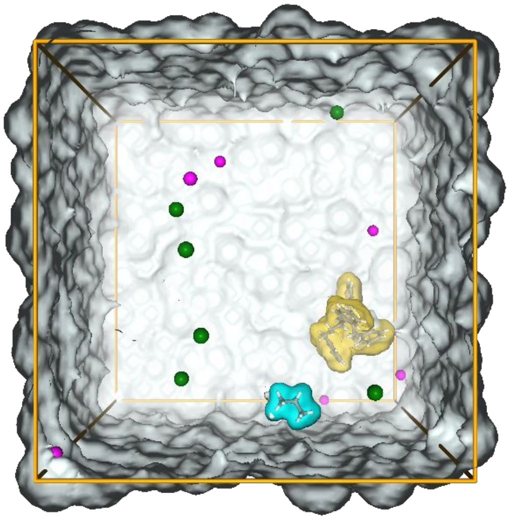

Figure S2. The example of a simulation box for parameterizing polymer – SCA interaction. In this case a polystyrene fragment (in yellow) represented by a tetramer is shown to interact with the valine side chain (in cyan). Both molecules were placed in the box with a side of 5.0 nm , solvated by TIP3P water, and neutralised by 0.15M KCL.

References.

1. Shi, R., Qian, H. J., & Lu, Z. Y. (2023). Coarse-grained molecular dynamics simulation of polymers: Structures and dynamics. *Wiley Interdisciplinary Reviews: Computational Molecular Science*, *13*(6), e1683.

2. Kästner, J. (2011). Umbrella sampling. *Wiley Interdisciplinary Reviews: Computational Molecular Science*, *1*(6), 932-942.

3. Barducci, A., Bussi, G., & Parrinello, M. (2008). Well-tempered metadynamics: a smoothly converging and tunable free-energy method. *Physical review letters*, *100*(2), 020603.

4. Hub, J. S., De Groot, B. L., & Van Der Spoel, D. (2010). g_wham□ A Free Weighted Histogram Analysis Implementation Including Robust Error and Autocorrelation Estimates. *Journal of chemical theory and computation*, *6*(12), 3713-3720.

5. Invernizzi, M., & Parrinello, M. (2020). Rethinking metadynamics: from bias potentials to probability distributions. *The journal of physical chemistry letters*, *11*(7), 2731-2736.

6. Kanhaiya, K., Kim, S., Im, W. *et al.* Accurate simulation of surfaces and interfaces of ten FCC metals and steel using Lennard–Jones potentials. *npj Comput Mater* **7**, 17 (2021).

7. Saeedimasine, M., Rahmani, R., & Lyubartsev, A. P. (2024). Biomolecular Adsorption on Nanomaterials: Combining Molecular Simulations with Machine Learning. *Journal of Chemical Information and Modeling*, *64*(9), 3799-3811.

8. Brandt, E. G., & Lyubartsev, A. P. (2015). Molecular dynamics simulations of adsorption of amino acid side chain analogues and a titanium binding peptide on the TiO2 (100) surface. *The Journal of Physical Chemistry C*, *119*(32), 18126-18139.

9. Jo, S., Cheng, X., Lee, J., Kim, S., Park, S. J., Patel, D. S., ... & Im, W. (2017). CHARMM-GUI 10 years for biomolecular modeling and simulation. *Journal of computational chemistry*, *38*(15), 1114-1124.

10. Subbotina, J., & Lobaskin, V. (2022). Multiscale modeling of bio-nano interactions of zero-valent silver nanoparticles. *The Journal of Physical Chemistry B*, *126*(6), 1301-1314.

11. Subbotina, J., Rouse, I., & Lobaskin, V. (2023). In silico prediction of protein binding affinities onto core–shell PEGylated noble metal nanoparticles for rational design of drug nanocarriers. *Nanoscale*, *15*(32), 13371-13383.
