# Supplementary material for: UANanoDock: A Web-Based UnitedAtom Multiscale Nanodocking Tool for Predicting Protein Adsorption onto Nanoparticles
Source: J Chem Inf Model. 2025 Mar 25;65(7):3142–53. doi: 10.1021/acs.jcim.4c02292 (PMC12004535; doi:10.1021/acs.jcim.4c02292)
Supplement: Supplementary file 3 — ci4c02292_si_003.pdf [file ci4c02292_si_003.pdf]

Moda powered by **Enalos Cloud Platform**

MODA for

UANanoDock

Simulated in project

UANanoDock

OVERVIEW of the SIMULATION			
1	**USER CASE**	UANanoDock	
2	**CHAIN OF MODELS**	**Data Transformation 1**<br><br>**Model 2**	PropKa<br>Data Transformation<br>UnitedAtom<br>Physics based Model
3	**PUBLICATION PEER - REVIEWING THE DATA**	DOI provided: Yes<br>DOI: https://www.enaloscloud.novamechanics.com/compsafenano/uananodock/	
4	**ACCESS CONDITIONS**	Access type: Free<br>Owner of workflow: UCD/NovaMechanics<br>Workflow access link: UANanoDock: A web-based United Atom multiscale nano-docking tool for predicting protein adsorption onto nanoparticles developed by Julia Subbotina, Panagiotis D. Kolokathis, Andreas Tsoumanis, Nikolaos K. Sidiropoulos, Ian Rouse, Iseult Lynch, Vladimir Lobaskin, and Antreas Afantitis, 2024 (Journal of Chemical Information and Modeling), NPCoronaPredict: A Computational Pipeline for the Prediction of the Nanoparticle–Biomolecule Corona by Ian Rouse, David Power, Julia Subbotina, and Vladimir Lobaskin. Journal of Chemical Information and Modeling, 2024. https://pubs.acs.org/doi/full/10.1021/acs.jcim.4c00434 Coarse-grained model of adsorption of blood plasma proteins onto nanoparticles by Hender Lopez and Vladimir Lobaskin. Journal of Chemical Physics, 2015. https://pubs.aip.org/aip/jcp/article/143/24/243138/965468/Coarse-grained-model-of-adsorption-of-blood-plasma	
5	**WORKFLOW AND ITS RATIONALE**	PropKa is used to calculate the charge of the amino acids (e.g., protonated amino acids are positively charged) which are used as input to UnitedAtom to describe the electrostatic interactions of the nanoparticle with the protein.	

Workflow picture

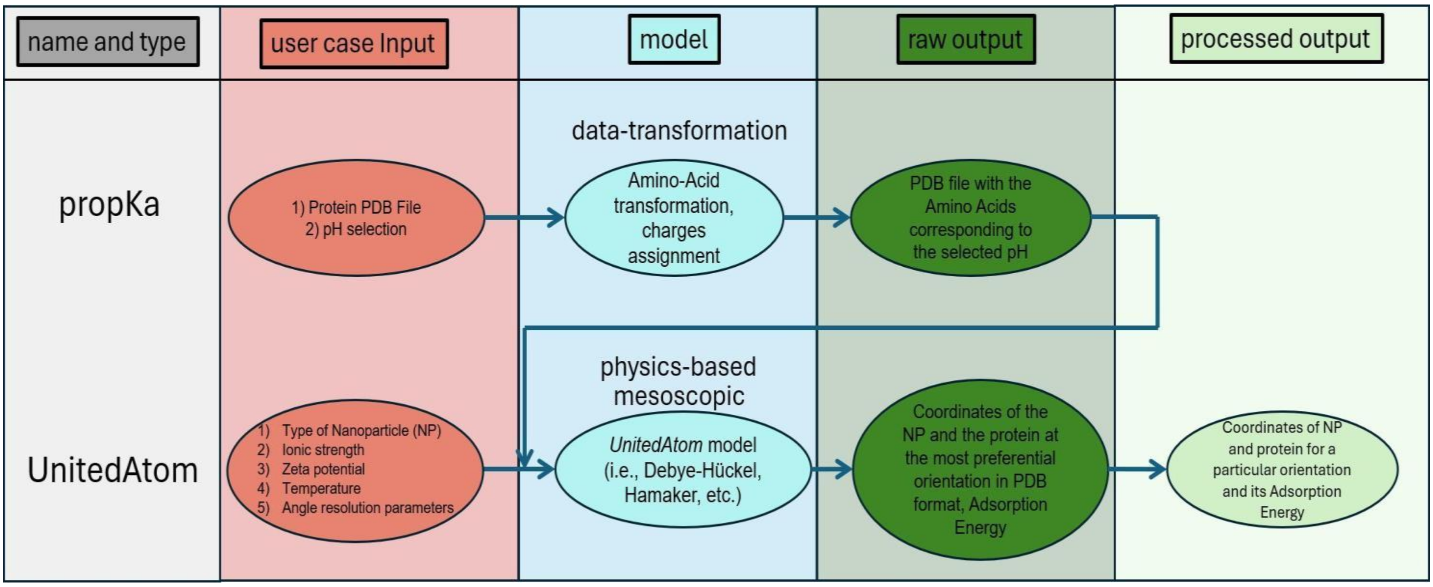

Each physics-based model used in this simulation is documented in four chapters as follows:

1. Aspect of the User Case or system simulated with this model
2. Model: Please ensure that the notions in the Physics Equation (PE) and Materials Relation (MR) are explained in a way that can be easily understood.
   - Tightly coupled models can be written up collectively in one set of four tables. To solve tightly coupled PEs one matrix is set up and solved in one go.
   - For continuum models the PE is often the conservation equations coded up in commercial (paid for) software packages.
   - Often the MR is established by the modeller.
3. Computational aspects include also a documentation of how the user case specifications are translated into computer language.
4. Post processing documents to describe how the raw output of one simulation is processed into the input for the next simulation. This information, given under section 4.1 in the first model, will be the same as the "simulated input" information under section 2.4 for the next model. This is the essence of model interoperability!

Pre-processing before the first model can be depicted in pink as it is considered to be part of the user-case.

Each data-based model in this simulation is documented in three chapters as follows:

1. Aspect of the User Case or system simulated with this data-based model
2. Data-based Model
3. Computational detail of the data-mining operation.

Data Transformation 1

PropKa

Aspect of the User Case/System		
**1.1**	**Aspect of the User Case to be simulated**	Protonate/deprotonate amino acids according to the pH of the surroundings.
**1.2**	**Material**	Proteins, Amino-acids
**1.3**	**Geometry**	Proteins dissolved in aquatic medium such as blood. Their size depends on the protein and is usually on the order of nanometres (nm).
**1.4**	**Time Lapse**	**Protonation/deprotonation** is a phenomenon that lasts some femtoseconds in the majority of the cases.
**1.5**	**Manufacturing process or in-service conditions**	Proteins can be natural (i.e., they come from living organisms) or artificially synthesized in laboratories
**1.6**	**Publication on this data**	Mats H. M. Olsson, Chresten R. Søndergaard, Michal Rostkowski, Jan H. Jensen, J. Chem. Theory Comput. 2011, 7, 2, 525–537, https://doi.org/10.1021/ct100578z

Data Transformation			
**2.0**	**Equation type and name**	The Henderson-Hasselbalch equation, pH = pKa + log([A–]/[HA])	
**2.1**	**Database and type**	List of Ka constants of aminoacids is provided in https://pubs.acs.org/doi/suppl/10.1021/ct100578z/suppl_file/ct100578z_si_001.pdf	
**2.2**	**Equation**	Hypothesis	If pH>pKa, the deprotonated amino acid appears. Otherwise, the protonated amino acid appears.
		Physical quantities	pH, acid dissociation constant (Ka)

Computational detail		
**3.1**	**Numerical Operations**	Change of amino acid name
**3.2**	**Software tool**	https://github.com/jensengroup/propka
**3.3**	**Margin Of Error**	Root Mean Square Deviation (rms) 0.79 (0.91) for Asp and Glu, 0.75 (0.97) for Tyr, 0.65 (0.72) for Lys, and 1.00 (1.37) for its residues

Model 2

UnitedAtom

Aspect of the User Case/System to be Simulated		
**1.1**	**Aspect of the User Case to be simulated**	Adsorption of Proteins onto Nanoparticle Surface
**1.2**	**Material**	Proteins, Nanoparticles in aquatic dispersion medium
**1.3**	**Geometry**	Spherical Nanoparticles and Proteins of various sizes and shapes
**1.4**	**Time Lapse**	The usual time lapse for the adsorption of a protein onto a nanoparticle surface is within the range of nanoseconds if it is assumed that nanoparticles and proteins are dissolved in the aquatic medium.
**1.5**	**Manufacturing process or in-service conditions**	Nanoparticles and proteins dissolved in aquatic dispersion medium
**1.6**	**Publication on this data**	https://pubs.acs.org/doi/full/10.1021/acs.jcim.4c00434<br>https://pubs.aip.org/aip/jcp/article/143/24/243138/965468/Coarse grained-model-of-adsorption-of-blood-plasma

Generic Physics Of The Model Equation			
**2.0**	**Model type and name**	Mesoscopic	
**2.1**	**Model entity**	grains, United Atoms	
**2.2**	**Model Physics/ Chemistry equation PE**	Equation	van der Waals interactions are described using the Hamaker equation after the assignment of a specific radius for each amino acid and a specific Hamaker constant (see https://doi.org/10.1088/1361-651X/ab3b6e for more details). At shorter distances between amino acids and the nanoparticle, the Hamaker potential is corrected to include the atomistic effects of the interactions.<br><br>For electrostatic interactions the Debye-Huckel potential is used, by providing the zeta potential of the nanoparticle in the dispersion medium.<br><br>Protein is treated as a rigid body.
		Physical quantities	potential energy, zeta potential, adsorption energy
**2.3**	**Materials relations**	Relation	Each amino acid is treated as a sphere having its center at the position of the alpha-carbon of the amino acid (see https://doi.org/10.1088/1361-651X/ab3b6e)
		Physical quantities /descriptors for each MR	distance of amino acid from nanoparticle surface,<br>charge of the amino acid,<br>zeta potential of the nanoparticle
**2.4**	**Simulated input**	**Protein data bank (**PDB) file of the protein, United Atom config file	

Solver and Computational translation of the specifications			
**3.1**	**Numerical Solver**	For each selected orientation, the adsorption Energy along the distance of the nearest amino acid from the nanoparticle's surface using a delta step (i.e., 0.003906 for UANanoDock user case) is calculated. The integral of the Adsorption Energy along the distance from the nanoparticle surface is calculated using finite differences. The upper limit of the integral is the 2.0 nm distance.	
**3.2**	**Software tool**	[opensource]<br>UnitedAtom (https://github.com/ucd-softmatterlab/NPCoronaPredict)<br>[free-web application]<br>UANanoDock<br>(https://www.enaloscloud.novamechanics.com/compsafenano/uananodock/)	
**3.3**	**Time step**	no timestep	
**3.4**	**Computational Representation**	Physics Equation,<br>Material Relations,<br>Material	see Section 2.2<br>The effect of solvent has been included implicitly through the equations and their parameters
**3.5**	**Computational boundary conditions**	non-periodic in X, Y and Z directions	
**3.6**	**Additional Solver Parameters**	UnitedAtom approach: Parameters and the values used in the config file for the UANanoDock user case:<br>enable-surface # defines if surface correction will be applied<br>enable-core # defines if Hamaker approximation will be used for the core of the nanoparticle<br>enable-electrostatic # defines if the electrostatics will be included in the calculation<br>simulation-steps = 2000 #<br>potential-cutoff = 5.0 nm # Cutoff of the Hamaker<br>potential-size = 1000 #<br>angle-delta = 5.0 # angle step for which the adsorption energy is calculated<br>num-random-samples = 6 # number of nearest angles calculations that will be assigned to a specific orientation<br>confirm-override-angle # information about UnitedAtom output file (*.uam)<br>bjerum-length # calculated automatically in UANanoDock	

Solver and Computational translation of the specifications		
		debye-length # calculated automatically in UANanoDock<br>temperature<br>zeta-potential<br>pdb-jitter-magnitude = 0 # uses uniform random numbers to define the angles that will be assigned as numerical average to the angle in the heatmap<br>pmf-cutoff = 1.2 # cutoff of the potential of mean force

Post processing

The “raw output” calculated by the model consists, per definition, of values for the physics variable in the PE(s). This variable is already specified in section 2.2 and this raw output will appear in the dark green circle in the workflow picture.

- to calculate values for physics variables for different entities of the next model. E.g. the output can be homogenised for larger volumes
- in the form of a MR for the next model
- into a Descriptor Rule that is the final output of the total simulation.

This processed output will appear in the light green circle in the workflow picture and also in section 2.4 of the next model (if there is one).

The methodology (often including physics) used to do this post processing calculation is documented in section 4.2.

Solver and Computational translation of the specifications		
**4.1**	**The processed output**	Average Adsorption Energy per protein orientation
**4.2**	**Methodologies**	Average
**4.3**	**Margin Of Error**	Standard deviation is included in the United Atom file
